# The gamma-aminobutyric acid type B (GABA_B_) receptor agonist baclofen inhibits morphine sensitization by decreasing the dopamine level in rat nucleus accumbens

**DOI:** 10.1186/1744-9081-8-20

**Published:** 2012-07-10

**Authors:** Zhenyu Fu, Hongfa Yang, Yuqiang Xiao, Gang Zhao, Haiyan Huang

**Affiliations:** 1 Department of Neurosurgery, First Hospital of Jilin University, ChangChun, 130021, China; 2 Department of Neurosurgery, The Daqing Oilfield General Hospital, Heilongjiang Province, DaQing, 5805999, China

**Keywords:** GABA receptor, Morphine, Sensitization, Dopamine, Nucleus accumbens, Baclofen

## Abstract

**Background:**

Repeated morphine exposure can induce behavioral sensitization. There are evidences have shown that central gamma-aminobutyric acid (GABA) system is involved in morphine dependence. However, the effect of a GABA_B_ receptor agonist baclofen on morphine-induced behavioral sensitization in rats is unclear.

**Methods:**

We used morphine-induced behavioral sensitization model in rat to investigate the effects of baclofen on behavioral sensitization. Moreover, dopamine release in the shell of the nucleus accumbens was evaluated using microdialysis assay in vivo.

**Results:**

The present study demonstrated that morphine challenge (3 mg/kg, s.c.) obviously enhanced the locomotor activity following 4-day consecutive morphine administration and 3-day withdrawal period, which indicated the expression of morphine sensitization. In addition, chronic treatment with baclofen (2.5, 5 mg/kg) significantly inhibited the development of morphine sensitization. It was also found that morphine challenge 3 days after repeated morphine administration produced a significant increase of extracellular dopamine release in nucleus accumbens. Furthermore, chronic treatment with baclofen decreased the dopamine release induced by morphine challenge.

**Conclusions:**

Our results indicated that gamma-aminobutyric acid system plays an important role in the morphine sensitization in rat and suggested that behavioral sensitization is a promising model to study the mechanism underlying drug abuse.

## Background

Repeated morphine administration can induce the neurochemical effects including mainly protein components and neurotransmission adaptations in the brain, which results in behavioral response underlying opioid dependence [1]. There are abundant reports have shown that locomotion sensitization has been suggested to mimic the brain changes that occur in the human addict [1-3], so it is intriguing animal model used to study neural mechanisms related to opioid dependence [4,5]. Morphine administration can produce a robust enhancement of locomotor activity in mice [6,7]. However, it induced dramatically an initial decrease and then an increase in locomotor activity in rats [1,8]. Hence, investigation of sensitization in rats may be interesting to better understand the potential mechanisms resulting in opioid dependence.

There are increasing findings have demonstrated that the mesolimbic dopamine (DA) system especially projecting from the ventral tegmental area (VTA) of the midbrain to the nucleus accumbens(NAc) is definitely involved in opioid dependence [9-12]. Opioids can increase DAergic transmission to the NAc by inhibiting the GABAergic interneurons in the VTA [9,13,14]. Moreover, non-DAergic neurotransmitter systems have been recently found to be involved in opioid addiction such as glutamatergic system [15]. Recent evidence has indicated that the GABAergic system is closely related to the mesolimbic dopaminergic system and involved in the modulation of addictive behavior [16-19].

Abundant convincing evidences have revealed that the GABA_B_ receptor agonist can suppress many behavior changes induced by opioid and non-opioid [17]. It has been reported that GABA_B_ receptor agonist decreases the cocaine and nicotine self-administration [20,21], blocks expression and sensitization of anxiety-like behavior induced by ethanol withdrawal [22], and suppresses the development of sensitization to the locomotor of amphetamine [23]. In addition, GABAergic potentiation following chronic morphine treatment attenuates the rewarding effects of opioids in the ventral tegmental area [24]. Above reports have indicated that gamma-aminobutyric acid transmission is more and more concerned.

Our current data focused on baclofen, a potent GABA_B_ receptor agonist, which was synthesized 30 years before a GABA_B_ receptor was found and possesses a high affinity for the GABA_B_ receptors and a strong intrinsic activity [17,25]. In the present study, we used the rat behavioral sensitization model to further study the effect of GABA_B_ receptor agonist on opioid dependence. The current study investigated the effect of baclofen on the development and expression of behavioral sensitization induced by morphine. Furthermore the effect of baclofen on extracellular DA release in NAc was also assessed.

## Methods

### Animals

Male Sprague–Dawley rats (Changchun Animal Center, China), weighing 250–270 g were used in the present study. All animals were housed individually in a temperature- and humidity-controlled room with food and water freely available under a 12-h light/dark cycle (lights on between 7:00 A.M. and 7:00 P.M.). Rats were acclimated to the housing conditions and handled daily for 3–4 days before the experiments began. All experiments were carried out during the light phase of the cycle. All animal use procedures were approved by the Jilin University Medical Center Animal Care and Use Committee and were conducted in accordance with the National Institutes of Health Guide for the Care and Use of Laboratory Animals.

### Drugs

Morphine hydrochloride was purchased from Qinghai Pharmaceutical Factory, China. Baclofen was obtained from Sigma (St. Louis, MO, USA). All drugs were dissolved with saline and injected in a volume of 1.0 ml/kg. Morphine and baclofen were given subcutaneously (s.c.). Sodium pentobarbital was given intraperitoneally (i.p.).

### The induction of morphine sensitization in rat

The apparatus for the measurement of locomotor activity consisted of eight chambers (60 × 60 × 40 cm). A video system was used to track the movement of the rats in each chamber for 3 h and the distance traveled was subsequently analyzed by software (Jiliang Software Company, Shanghai, China). The protocol for locomotion sensitization induced by morphine was based on previous studies [26]. For all tests, the rats were habituated to the chamber for 15 min prior to the s. c. injection. The protocol includes 4 days of morphine repeated treatment, followed by 3 days of withdrawal. Then, Morphine (3 mg/kg, s.c.) challenge testing was performed on day 8. Repeated treatment was as follows: On day 1, rats were habituated to chamber for 15 min, and then given morphine (10 mg/kg, s.c.). After the morphine injection, the rats were returned immediately to the chamber where locomotor activity was monitored for 3 h. On days 2, rats were given morphine (10 mg/kg, s.c.) twice daily. The first injection was given at 7:00 am after a 15-min habituation in the chambers, and then locomotor activity was monitored for 3 h, while the second morphine was given at 5:00 pm in the home cages. On days 3–4, rats were given morphine (20 mg/kg, s.c.) twice daily. The first injection and the second morphine injection had been described above. After the 3-day withdrawal period without treatment, rats were subjected to an acute challenge of morphine (3 mg/kg s.c.) to test the expression of behavioral sensitization on day 8. Two groups of rats were given morphine and saline (1 ml/kg, s.c.) for 4 consecutive days and their activity was measured for 3 h after each administration as described above.

### Effects of the GABAB receptor agonist baclofen on locomotor activity in rat

In order to study the effect of acute and chronic administration of baclofen on locomotor activity, four groups of rats were exposed to saline or baclofen (1.25, 2.5, 5 mg/kg, s.c.) for 8 continuous days. On days 1 and 8, the rats were administered saline or baclofen and then put into the test chambers to monitor the locomotor activity for 3 h.

### Effects of the GABA_B_ receptor agonist baclofen on the acute morphine stimulation of locomotion in rat

In order to study the effect of baclofen on acute morphine-induced locomotion, four groups of rats were exposed to morphine and one group of rats was given saline. For morphine treatment group, saline or baclofen (1.25, 2.5, 5 mg/kg, s.c.) was administered 15 min prior to the morphine injection. Locomotor activity was measured for 3 h on day 1 immediately after morphine administration.

### Effects of acute treatment of baclofen on the expression of morphine-induced sensitization

Four groups of rats were exposed to morphine and one group of rats was given saline for 4 consecutive days as described above. After a 3-day withdrawal period, all five groups of animals were subjected to an acute challenge of morphine (3 mg/kg, s.c.) on day 8. Four morphine-treated groups were pretreated with saline or baclofen (1.25, 2.5, 5 mg/kg, s.c.) 15 min prior to the morphine challenge. The locomotor activity was monitored for 3 h in the chambers.

### Effects of chronic treatment of baclofen on the development of morphine sensitization

Four groups of rats were exposed to morphine and one group of rats was given saline for 4 consecutive days. For morphine group, saline or baclofen (1.25, 2.5, 5 mg/kg, s.c.) was concomitantly administered 15 min prior to the morphine injection. During a 3-day withdrawal period, rats in morphine group were still injected with saline or baclofen (1.25, 2.5, 5 mg/kg, s.c.) daily. On day 8, all five groups of animals were subjected to an acute challenge of morphine (3 mg/kg, s.c.). Locomotor activity was measured for 3 h in the chambers immediately after morphine administration.

### In vivo microdialysis experiments

**Figure 1:**
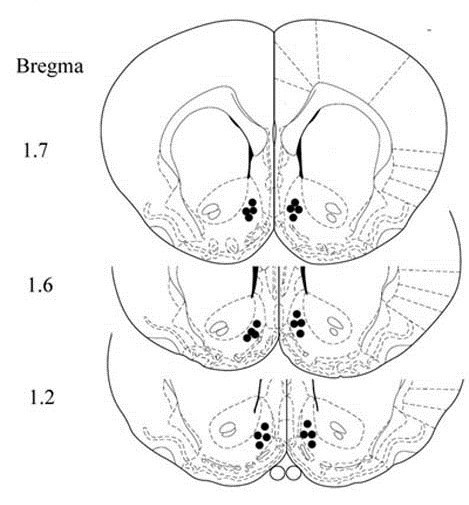
**Location of the microdialysis probe in the shell portion of the nucleus accumbens.** Schematic representation of the location of the dialysis probes in the shell portion of the nucleus accumbens for all rats included in the experiment.

The rats were anaesthetized with sodium pentobarbital (50 mg/kg, i.p.) and placed in a stereotaxic frame. The skull was exposed and one small hole was drilled for implantation of the microdialysis probes. Stereotaxic coordinates for the NAc shell were determined according to the previous report [27]. The coordinates were anteroposterior (AP) 1.7 mm from the bregma, mediolateral (ML) ± 0.6 mm, and dorsoventral (DV) −6.0 mm from the surface of the skull (Figure 1). The microdialysis probe was perfused with sterilized artificial cerebrospinal fluid (in mM): NaCl 147, KCl 4, CaCl2 2.2, pH 7.4 [26]. The microdialysis samples were collected by the microsyringe pump (ESP-64, Eicom, Japan) at the microdialysis rate of 1.5 μl/min every 20 min after 1 h of equilibration. DA and its metabolites in dialysates were measured by high-performance liquid chromatography (HPLC) using electrochemical detection with a glassy carbon electrode. The mobile phase consisted of 85 mM citrate, 100 mM sodium acetate, 0.9 mM octyl-sodium sulfate, 0.2 mM EDTA, and 15% methanol, pH 3.7. The mobile phase was pumped at 600 ul/min with a Lachrom system (Eicom-Hitachi, Japan) and the separation was performed with a microbore reverse-phase column (DIKMA Technologies Ltd., Beijing, China).The volume of injection was 50 μl.

The treatment protocol was as described above. Implantation of the microdialysis probes was performed during the 3-day morphine withdrawal period. On day 8, following the basal levels of DA became stable, all rats were given a challenge dose of morphine (3 mg/kg, s.c.) and the dialysates were monitored for 3 h. At the end of each experiment, rats were sacrificed by overdose injection of pentobarbital sodium, and their brains were fixed in 10% neutral-buffered formalin. The location of the dialysis probes was verified histologically with cresyl violet staining.

### Statistical analysis

Data are expressed as mean ± S.E.M. Student’s t-test was used to compare the difference between two experimental groups, and one- way analysis of variance (ANOVA) was used to compare the differences among three or more groups, followed by Newman-Keuls Multiple Comparison Test. For induction of morphine sensitization, Data were analyzed with a repeated two- way ANOVA and Post hoc tests were conducted using Bonferroni tests; For the microdialysis experiments, the average of the last three samples was considered to be the baseline and defined as 100%; All statistical tests were two-tailed and significance was defined as p < 0.05.

## Results

### Repeated morphine treatment induced behavioral sensitization in rat

**Figure 2:**
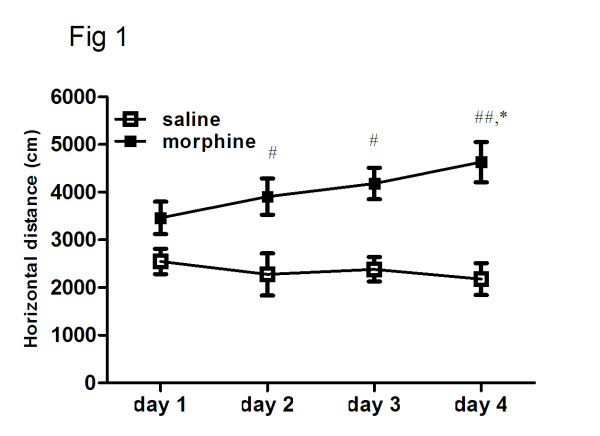
**The induction of behavioral sensitization of morphine in rat.** Two groups of rats were exposed to morphine or saline for 4 consecutive days. Rats were given morphine or saline once daily on day 1 and twice daily on days 2–4 as described previously. The locomotor activity was monitored for 3 h in the chambers after the first injection once daily. Each point represents the total distance traveled for the 3 h test period. Data are shown as means ± S.E.M (n = 8 per group). ^*^ P < 0.01 compared to the first administration within group; ^#^ P < 0.05 and ^##^ P < 0.01 compared to the corresponding saline control group.

Two groups of rats were given saline or morphine for 4 consecutive days as described above and their locomotor activity was recorded for 3 h after the first injection once daily. As shown in Figure 2, after 4 -day consecutive morphine administration, Repeated one-way ANOVA revealed that morphine treatment for 4 days significantly enhance the locomotor activity in rats (F(3, 21) = 3.21, P <0.05). Newman-Keuls multiple comparison test indicated that the locomotor activity differed significantly between day 1 and day 4 in morphine treatment group. (q = 4.28, P < 0.05). No significant difference was found in saline treatment group (F(3, 21) = 0.32, P =0.81).

The repeated two-way ANOVA revealed that the locomotor activity was dependent on the drug treatment (F(1, 42) = 13.67, P < 0.01), days (F(3, 42) = 0.64, P =0.59) and the interaction between the drug treatment and the days (F(3, 42) = 1.96, P =0.13). Bonferroni post hoc tests indicated that repeated morphine treatment significantly increase locomotor activity of rat compared to the corresponding saline control group on day 2 (t = 2.69, P < 0.05), day 3 (t = 2.98, P < 0.05) and day 4 (t = 4.07, P < 0.01).

### Effects of baclofen on locomotor activity in rat

**Figure 3:**
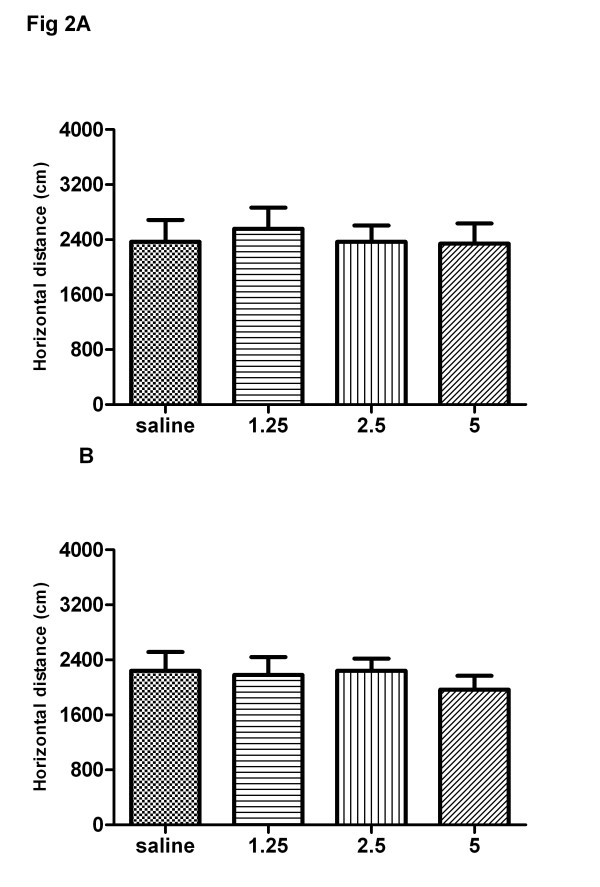
**Effects of acute and chronic administration of baclofen on locomotor activity in rat.** Rats were exposed to saline or baclofen (1.25, 2.5, 5 mg/kg, s.c.) for 8 continuous days. On days 1(**a**) and 8 (**b**), the rats were administered saline or baclofen and then put into the test chambers to monitor the locomotor activity for 3 h. Data are expressed as the means ± S.E.M. (n = 8 per group).

As shown in Figure 3a, one-way ANOVA revealed that acute treatment with baclofen (1.25, 2.5, 5 mg/kg, s.c.) had no significant effect on locomotor activity in rat (F(3, 28) = 0.118, P > 0.05) (Figure 3a). After 8 continuous days of treatment with saline or baclofen (1.25, 2.5, 5 mg/kg, s.c.), On day 8, the rats were administered saline or baclofen and then put into the test chambers to monitor the locomotor activity for 3 h. As shown in Figure 3b, one-way ANOVA revealed that the locomotor activity of the baclofen-treated groups showed no significant difference compared to the saline group (F(3, 28) = 0.323, P > 0.05) (Figure 3b). High dose of baclofen (5 mg/kg) decreased the locomotion activity, but Newman-Keuls multiple comparison test indicated that there is no significant difference compared to the saline group(q = 2.35, P > 0.05).

### Effects of baclofen on the acute morphine stimulation of locomotion in rat

**Figure 4:**
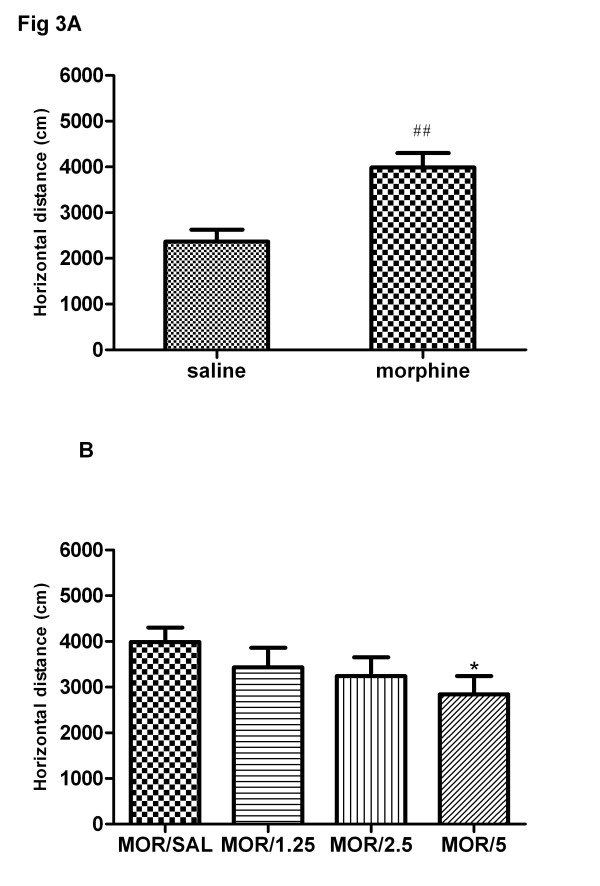
**Effects of single administration of baclofen on the acute morphine stimulation of locomotor activity.** Four groups of rats were exposed to morphine and one group of rats was given saline. For morphine treatment group, saline or baclofen (1.25, 2.5, 5 mg/kg, s.c.) was administered 15 min prior to the morphine injection (**b**). Locomotor activity was measured for 3 h on day 1 immediately after morphine administration. Each bar represents the total distance traveled for the 3 h test period. **a**: the saline/saline group and morphine/saline group. Data are shown as means ± S.E.M (n = 8 per group). ^##^ P < 0.01 compared to saline/saline group; ^*^ P < 0.05 compared to morphine/saline group.

In order to study the effect of baclofen on acute morphine-induced locomotion, rats pretreated with saline or baclofen (1.25, 2.5, 5 mg/kg) and then challenged with morphine on day 1. As shown in Figure 4a, morphine treatment on day 1significantly enhanced locomotor activity in rats compared to the saline control group (t = 3.98, P < 0.01). One-way ANOVA revealed that baclofen pretreatment inhibited the enhancement of acute morphine stimulation of locomotion (F(4, 35) = 2.95, P < 0.05) (Fig. b). Newman-Keuls multiple comparison test indicated that baclofen (5 mg/kg) significantly affect the enhancement of sensitization compared to the morphine/saline group (q = 4.2, P < 0.05). There was no significant difference in the locomotor activity between saline/morphine and baclofen (1.25, 2.5 mg/kg)/morphine groups.

### Effects of acute treatment of baclofen on the expression of morphine-induced sensitization

**Figure 5:**
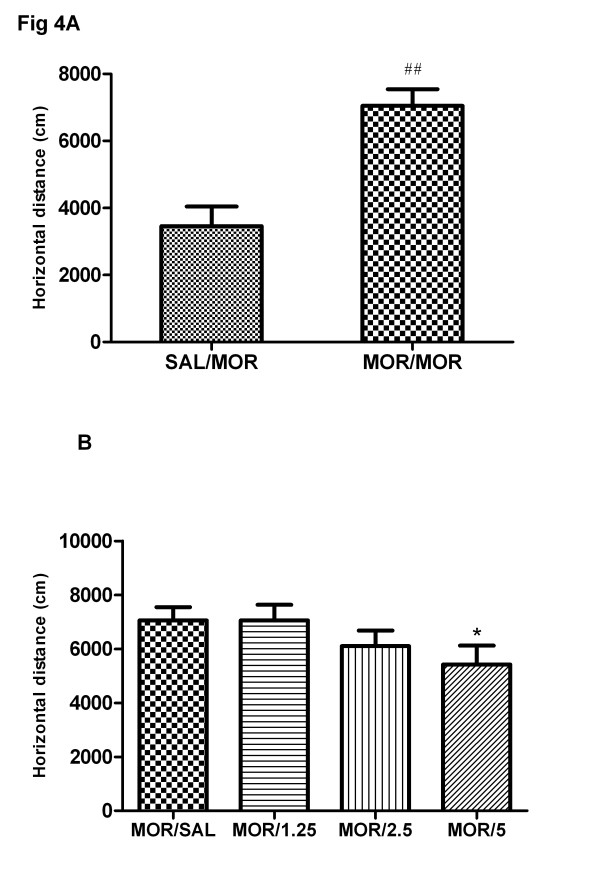
**Effects of acute treatment of baclofen on the expression of morphine-induced sensitization.** Four groups of rats were exposed to morphine and one group of rats was given saline for 4 consecutive days as described previously. After a 3-day withdrawal period, all five groups of animals were subjected to an acute challenge of morphine (3 mg/kg, s.c.) on day 8. Four morphine-treated groups were pretreated with saline or baclofen (1.25, 2.5, 5 mg/kg, s.c.) 15 min prior to the morphine challenge (**b**). The locomotor activity was monitored for 3 h in the chambers. Each bar represents the total distance traveled for the 3 h test period. **a**: the saline/morphine group and morphine/saline group. Data are shown as means ± S.E.M (n = 7–8 per group). ^##^ P < 0.01 compared to saline/morphine group; ^*^ P < 0.05 compared to morphine/saline group.

After 4 days of repeated morphine treatment and 3 days of withdrawal, all rats were challenged with morphine (3 mg/kg) on day 8. As shown in Figure 5a, morphine challenge induced significantly the expression of behavioral sensitization (t = 4.68, P < 0.01) in the repeated morphine treatment group compared to saline control group. One-way ANOVA showed that acute baclofen pretreatment 15 min before morphine challenge dose-dependently inhibited the expression of morphine-induced behavioral sensitization [F(4,35) = 6.32, P < 0.01] (Figure 5b).

Newman-Keuls multiple comparison test indicated that baclofen (5 mg/kg) significantly inhibited the expression of morphine-induced behavioral sensitization (q = 3.31, P < 0.05), while baclofen (1.25, 2.5 mg/kg) did not affect significantly the magnitude of sensitization (P > 0.05).

### Effects of chronic treatment of baclofen on the development of morphine sensitization

**Figure 6:**
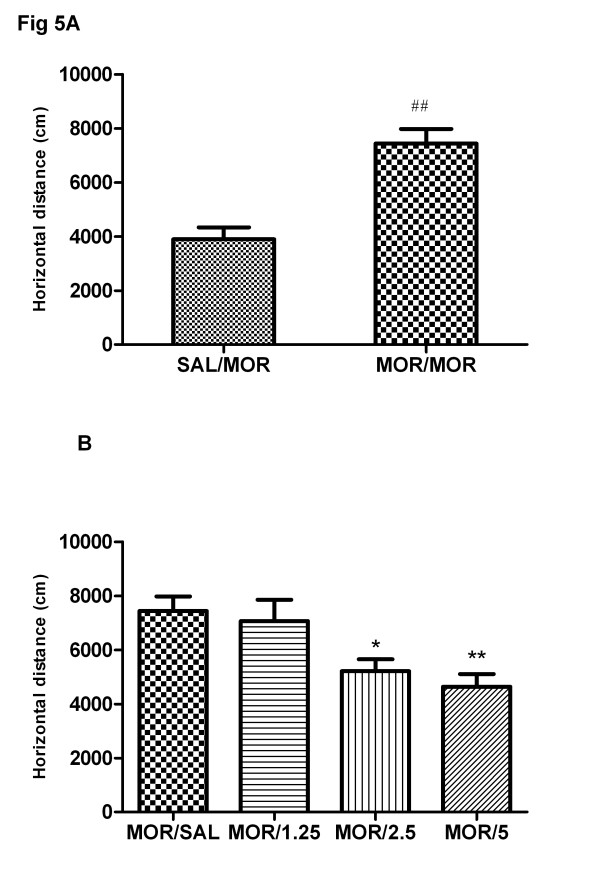
**Effects of chronic treatment of baclofen on the development of morphine sensitization.** Four groups of rats were exposed to morphine and one group of rats was given saline for 4 consecutive days. For morphine group, saline or baclofen (1.25, 2.5, 5 mg/kg, s.c.) was concomitantly administered 15 min prior to the morphine injection. During a 3-day withdrawal period, rats in morphine group were still injected with saline or baclofen (1.25, 2.5, 5 mg/kg, s.c.) daily (**b**). On day 8, all five groups of animals were subjected to an acute challenge of morphine (3 mg/kg, s.c.). The locomotor activity was monitored for 3 h in the chambers. Each bar represents the total distance traveled for the 3 h test period. **a**: the saline/morphine group and morphine/saline group. Data are shown as means ± S.E.M (n = 8 per group). ^##^ P < 0.01 compared to saline/morphine group; ^*^ P < 0.05 and ^**^ P < 0.01 compared to morphine/saline group.

As shown in Figure 6a, morphine challenge induced significantly the expression of behavioral sensitization (t = 5.07, P < 0.01) in the repeated morphine treatment group compared to saline control group. One-way ANOVA showed that chronic baclofen treatment dose-dependently inhibited the development of morphine-induced behavioral sensitization [F(4,35) = 7.75, P < 0.01] (Figure 6b). Newman-Keuls multiple comparison test indicated that chronic baclofen (2.5, 5 mg/kg) significantly inhibited the development of morphine-induced behavioral sensitization (q = 2.85, P < 0.05; q = 3.59, P < 0.01), while baclofen (1.25 mg/kg) did not affect significantly the magnitude of sensitization (P > 0.05).

### Effects of acute treatment of baclofen on morphine challenge-induced release of dopamine in the NAc of rats

**Figure 7:**
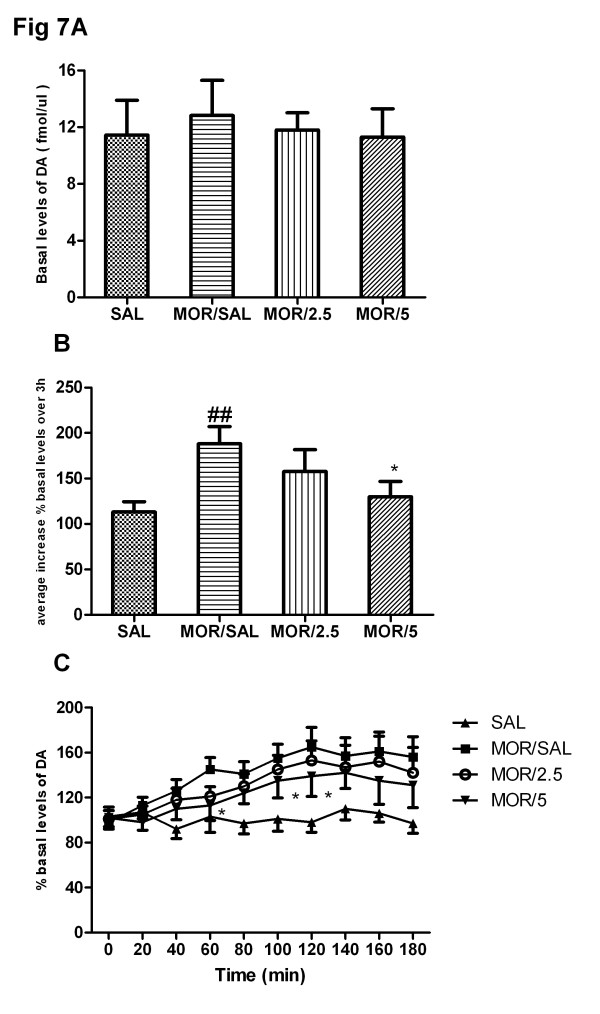
**Effects of acute treatment of baclofen on morphine challenge-induced release of dopamine in the NAc in rat.** Drug treatments are as described previously. After 4-day repeated morphine administration, implantation of the microdialysis probes was performed on day 5. On day 8, after basal levels of DA and became stable (**a**), all rats were subjected to an acute challenge of morphine (3 mg/kg, s.c.) . The dialysates were monitored for 3 h. DA release was expressed as average increase of% basal level over 3 h (**b**) and percentages of basal mean levels per 20-min intervals (**c**). ^##^ P < 0.01, compared to saline/morphine control group; ^*^ P < 0.05 compared to the morphine/saline treatment group; n = 8 per group.

As shown in Figure 6a, acute baclofen treatment did not effect the extracellular DA level in NAc before the final morphine challenge [F(3,28) = 0.105, P = 0.349]. Figure 7b indicated that morphine challenge significantly enhanced extracellular DA release in NAc in repeated morphine administration compared to the saline control group (t = 3.52, P < 0.01). Moreover, One-way ANOVA showed that acute baclofen treatment significantly attenuated an increase of extracellular DA release in NAc induced by morphine challenge [F(3,28) = 3.12, P < 0.05]. The effects of acute baclofen treatment on DA release in 20 min interval were shown in Figure 7c, the repeated two-way ANOVA revealed that the extracellular DA release in NAc was dependent on the drug treatment (F(3, 252) = 9.36, P < 0.05), times (F(9, 252) = 12.29, P < 0.01) and the interaction between the drug treatment and the times (F(27, 252) = 8.66, P < 0.05). Bonferroni post hoc tests indicated that acute baclofen (5 mg/kg) treatment significantly decrease DA release compared to the corresponding saline control group on 60 min (t = 2.84, P < 0.05), 100 min (t = 3.15, P < 0.05) and 120 min (t = 2.91, P < 0.05). Moreover, baclofen (2.5 mg/kg) pretreatment attenuated extracellular DA release in NAc and yet had no significant effect.

### Effects of chronic treatment of baclofen on release of dopamine in the NAc of rats

**Figure 8:**
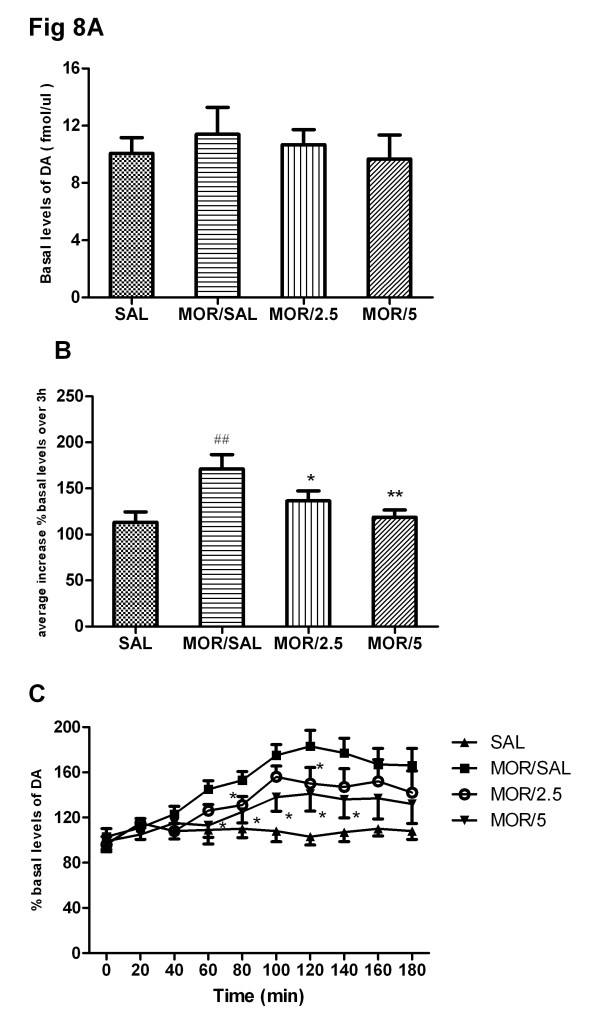
**Effects of chronic treatment of baclofen on morphine challenge-induced release of dopamine in the NAc in rat.** Drug treatments are as described previously. After 4-day repeated morphine administration, implantation of the microdialysis probes was performed during the 3-day morphine withdrawal period. On day 8, after basal levels of DA and became stable (**a**), all rats were subjected to an acute challenge of morphine (3 mg/kg, s.c.).The dialysates were monitored for 3 h. DA release was expressed as average increase of% basal level over 3 h (**b**) and percentages of basal mean levels per 20-min intervals (**c**). ^##^ P < 0.01, compared to saline/morphine control group; ^*^ P < 0.05 and ^**^ P < 0.01, compared to the morphine/saline treatment group; n = 7–8 rats per group.

As shown in Figure 8a , repeated morphine administration did not change the extracellular DA level in NAc before the final morphine challenge compared to saline control group (t = 0.62, P =0.53). One-way ANOVA showed that chronic baclofen treatment did not effect the extracellular DA level in NAc before the final morphine challenge [F(3,27) = 0.26, P = 0.42]. Figure 8b indicated that morphine challenge significantly enhanced extracellular DA release in NAc in repeated morphine administration compared to the saline control group (t = 3.06, P < 0.01). Moreover, One-way ANOVA showed that chronic baclofen treatment significantly attenuated an increase of extracellular DA release in NAc induced by morphine challenge [F(3,27) = 4.95, P < 0.01]. Newman-Keuls multiple comparison test indicated that chronic baclofen (2.5, 5 mg/kg) significantly suppressed extracellular DA release in NAc after morphine challenge (q = 2.96, P < 0.05; q = 4.51, P < 0.01). The effects of chronic baclofen treatment on DA release in 20 min interval were shown in Figure 8c, the repeated two-way ANOVA revealed that the extracellular DA release in NAc was dependent on the drug treatment (F(3, 251) = 12.17, P < 0.01), times (F(9, 251) = 15.31, P < 0.01) and the interaction between the drug treatment and the times (F(27, 251) = 10.62, P < 0.01). Bonferroni post hoc tests indicated that acute baclofen (5 mg/kg) treatment significantly decrease DA release compared to the corresponding saline control group on 60 min (t = 3.26, P < 0.05), 80 min (t = 2.97, P < 0.05) , 100 min (t = 3.67, P < 0.05), 120 min (t = 2.98, P < 0.05) and 140 min (t = 3.91, P < 0.05). Moreover, baclofen (2.5 mg/kg) pretreatment attenuated extracellular DA release in NAc on 80 min (t = 2.79, P < 0.05) and 120 min (t = 3.05, P < 0.05).

## Discussion

In recent years, GABA_B_ receptor agonist has received a considerable research interest for its extensive therapeutic implications associated with alcohol dependence [28], cocaine abuse [20,29], nicotine [30,31] and opiates addiction [32-34]. An involvement of GABA_B_ receptor agonist in modulating dopaminergic effects related to opiates abuse have been reported by several previous studies [18,34,35], which is encouraging to consider the possibility that GABA_B_ receptor agonist could play an important role in opiates addiction.

Baclofen, a potent GABA_B_ receptor agonist was determined to attenuate morphine discriminative stimulus effects [32], inhibit morphine withdrawal signs [36,37] and decreased morphine-induced hyperactivity in mice [38]. However, its potential role in morphine sensitization in rats still remains to investigate further. From this perspective, in the present work, we determined to evaluate that the effect of baclofen on the development and expression of morphine-induced behavioral sensitization. In addition, extracellular DA release in NAc was also detected.

Baclofen reversed the behavioral sensitization to morphine in rats had been reported [39]. However, in our study, we systemically evaluated the effect of baclofen on development and expression of morphine sensitization and dopamine release in NA in rats. The main finding of the present work is that repeated baclofen administration during 4-day consecutive morphine administration and 3-day withdrawal period inhibits the acquisition and expression of morphine sensitization and dopamine release in the shell of NAc, which are consistent with previous reports that the shell of NAc plays a critical role in the behavioral sensitization [27,40].

Repeated administration of opiates can lead to increased locomotor activity which is called behavioral sensitization [41-44]. It has been widely recognized that behavioral sensitization plays an important role in drug addiction such as relapse [45,46], compulsive drug-seeking [47-49]. Based on the crucial role of behavioral sensitization in drug addiction, it is necessary to investigate the effect of baclofen on morphine-induced behavioral sensitization which can also provide new insights into the understanding of potential mechanism underlying the morphine dependence. The present study confirmed that repeated morphine administration for 4 days in rat produces an increase in locomotion activity in response to an acute challenge of morphine following 3 days of withdrawal period, which was in agreement with preliminary studies [26,50], suggesting the development of locomotion sensitization. Furthermore, our current findings showed that chronic treatment with different doses of baclofen significantly inhibited the development of behavioral sensitization induced by morphine. In addition, acute treatment of only high dose of baclofen has effect on morphine sensitization. The present study provides the evidence that the increased GABA release is really suppressed morphine-induced sensitization which suggested the possibility that GABA transmission plays a key role in sensitization.

There are substantial findings revealed that mesolimbic DAergic pathway might be involved in modulating behavioral sensitization [51], especially the nucleus accumbens which receives DAergic projection arising from DA neurons in the ventral tegmental area (VTA) [11,13,14,52]. To detect the possible mechanism contribute to the inhibition of baclofen on behavioral sensitization following repeated exposure to morphine, we assessed the effect of baclofen on extracellular DA release in NAc. Results showed that, after 3 days withdrawal following repeated morphine administration, morphine challenge significantly increased extracellular DA release in NAc, consistent with the previous report [50]. It is also found that pretreatment with baclofen significantly prevents enhancement of extracellular DA release in NAc induced by morphine challenge.

The NAc includes a medioventral shell region and a dorsolateral core region and receive dopaminergic and GABAergic projections from the VTA and other regions [53]. Previous studies had shown that GABAB receptor activation in the VTA reduced the synaptic dopamine release in the NAc [7,53]. Therefore, Activation of GABAB receptors localized on dopaminergic neurons in the VTA might be involved in the effect of baclofen in our study which attenuated both the development and expression of morphine sensitization and dopamine release in the shell of NAc. Moreover, GABAB receptors have been found in the NAc and other region. The effect of baclofen on acquisition and expression of morphine sensitization might be also induced by GABAB receptor activation in the shell of NAc.

## Conclusions

Our results have demonstrated that acute morphine challenge induced behavioral sensitization after repeated morphine administration followed by 3 days withdrawal period. The fact that the GABA_B_ receptor agonist, baclofen, dose-dependently inhibited morphine sensitization by prevention of extracellular DA release in NAc has further confirmed that GABAergic neurotransmission could be participated in modulating morphine sensitization.

## Abbreviations

GABA: Gamma-aminobutyric acid; DA: Dopamine; VTA: Ventral tegmental area; NAc: Nucleus accumbens.

## Competing interests

The authors declare that they have no competing interests.

## Authors’ contributions

ZF and HY designed and performed the research and took part in the data analyses and manuscript preparation. YX and GZ contributed to the data analyses and manuscript preparation. HH took part in the design of the experiment and helped write the manuscript. All authors read and approved the final manuscript.
